# Clonal Confinement of a Highly Mobile Resistance Element Driven by Combination Therapy in Rhodococcus equi

**DOI:** 10.1128/mBio.02260-19

**Published:** 2019-10-15

**Authors:** Sonsiray Álvarez-Narváez, Steeve Giguère, Elisa Anastasi, Jack Hearn, Mariela Scortti, José A. Vázquez-Boland

**Affiliations:** a Microbial Pathogenesis Group, Infection Medicine, Edinburgh Medical School (Biomedical Sciences), University of Edinburgh, Edinburgh, United Kingdom; b Department of Large Animal Medicine, University of Georgia, Athens, Georgia, USA; c Institute of Evolutionary Biology, University of Edinburgh, Edinburgh, United Kingdom; Institut Pasteur

**Keywords:** IS*Re46*, MDR clonality, *Rhodococcus equi*, *Rhodococcus equi* MDR clone, *Rhodococcus hoagii*, *Rhodococcus* pneumonia, Tn*RErm46*, *erm*(46), macrolide resistance, multidrug resistance, pRErm46, rifampin resistance

## Abstract

Antibiotic use has been linked to changes in the population structure of human pathogens and the clonal expansion of multidrug-resistant (MDR) strains among healthcare- and community-acquired infections. Here we present a compelling example in a veterinary pathogen, Rhodococcus equi, the causative agent of a severe pulmonary infection affecting foals worldwide. We show that the *erm*(46) gene responsible for emerging macrolide resistance among equine R. equi isolates in the United States is part of a 6.9-kb transposable element, Tn*RErm46*, actively mobilized by an IS*481* family transposase. Tn*RErm46* is carried on an 87-kb conjugative plasmid, pRErm46, transferable between R. equi strains at frequencies up to 10^−3^. The *erm*(46) gene becomes stabilized in R. equi by pRErm46’s apparent fitness neutrality and wholesale Tn*RErm46* transposition onto the host genome. This includes the conjugally exchangeable pVAPA virulence plasmid, enabling the possibility of cotransfer of two essential traits for survival in macrolide-treated foals in a single mating event. Despite its high horizontal transfer potential, phylogenomic analyses show that *erm*(46) is paradoxically confined to a specific R. equi clone, 2287. R. equi 2287 also carries a unique *rpoB*^S531F^ mutation conferring high-level resistance to rifampin, systematically administered together with macrolides against rhodococcal pneumonia on equine farms. Our data illustrate that under sustained combination therapy, several independent “founder” genetic events are concurrently required for resistance, limiting not only its emergence but also, crucially, horizontal spread, ultimately determining multiresistance clonality.

## Abstract (precis)

MDR clades arise upon acquisition of resistance traits, but the determinants of their clonal expansion remain largely undefined. Taking advantage of the unique features of Rhodococcus equi infection control in equine farms, involving the same dual antibiotic treatment since the 1980s (a macrolide and rifampin), this study sheds light into the determinants of multiresistance clonality and the importance of combination therapy in limiting the dissemination of mobile resistance elements. Clinically effective therapeutic alternatives against R. equi foal pneumonia are currently lacking, and the identified macrolide-rifampin MDR clone 2287 has serious implications. Still at early stages of evolution and local spread, R. equi 2287 may disseminate globally, posing a significant threat to the equine industry and, also, public health due to the risk of zoonotic transmission. The characterization of the 2287 clone and its resistance determinants will enable targeted surveillance and control interventions to tackle the emergence of MDR R. equi.

## INTRODUCTION

Rhodococcus equi is a soil-dwelling facultative intracellular actinobacterium that causes pyogranulomatous infections in animals and immunocompromised people (1–3). While affecting a variety of animal species, R. equi is most commonly isolated from foals, in which it causes a life-threatening multifocal pneumonic disease with frequent extrapulmonary involvement (1, 3). Attack rates in farms where the disease is endemic are typically 10 to 20% or higher, and a larger proportion of foals can be subclinically affected (4, 5). Equine isolates harbor a host-adapted virulence plasmid of ≈80 kb designated pVAPA (6–9), required for intramacrophage survival and pathogenesis (10). pVAPA is easily lost in the absence of host selection but can be readily regained, and transmitted between isolates, via conjugal transfer (7, 9, 11–13). Maintenance of environmental pools of pVAPA-bearing (i.e., “virulent”) R. equi through fecal-oral cycling is thought to contribute to farm-level endemicity. Together with two other host-associated virulence plasmids (porcine pVAPB and ruminant pVAPN) (3, 8, 9, 13), pVAPA plasmids can be found in human isolates (7), consistent with equine settings being a potential source of human R. equi infection (2).

Control of foal rhodococcosis is challenging due to the lack of an effective vaccine and relies on antibiotic therapy (4, 14). Treatments are not only applied to clinically affected animals but also preventatively to presumptive cases identified by thoracic ultrasonographic screenings (4, 5, 15–17). Although R. equi is susceptible to a variety of antimicrobials *in vitro* (18, 19), many drugs are ineffective *in vivo* (2, 20). Inconsistent *in vitro* susceptibility to some antimicrobials is also observed, with intrinsic resistance noted for β-lactams and quinolones (18, 21–24). Thus, the mainstay of R. equi therapeutics on equine farms has been and remains the combination of a macrolide (erythromycin, clarithromycin, or azithromycin) and rifampin (4).

Since its introduction in the late 1980s (20, 25), the macrolide-rifampin combination has dramatically reduced foal mortality, until recently with little evidence of resistance (4, 26). A changing trend was, however, observed in the 2000s, with rates of up to ≈4% of high-level resistance to both macrolides (MICs, 12 to >256 μg/ml) and rifampin (MIC, ≥32 μg/ml) reported in the United States (28). Alarmingly, a recent study found up to 40% of foals yielding isolates highly resistant to macrolides and rifampin as a result of mass antimicrobial treatment (16). While rifampin-only resistance due to *rpoB* mutations has been reported for R. equi (19, 23, 27–30), including at least two instances documented during monotherapy in foals (30, 31), macrolide resistance has so far always been associated with rifampin resistance (16, 28, 32).

We recently identified a novel *erm* gene, *erm*(46), as the cause of the emerging macrolide resistance among equine R. equi isolates in the United States (32). *erm*(46) confers resistance against all macrolides (MIC_90_ 64 to >256 μg/ml for azithromycin, clarithromycin, and erythromycin), lincosamides, and streptogramins B (MLS). In contrast to the closely related mycobacterial *erm* rRNA methylase genes *erm*(38) and *erm*(39), which are part of the intrinsic core resistome, *erm*(46) was transferable by mating at frequencies of up to 10^−2^ to 10^−3^ (32). This was highly concerning not only due to the risk of rapid spread among equine-associated R. equi organisms but also because macrolides are critically important antibiotics in human medicine (33) and are used in the treatment of R. equi infections in people (2).

Here we report the detailed genetic characterization of the *erm*(46) determinant. *erm*(46) is part of a highly mobile transposable element carried on a conjugative plasmid, pRErm46, from which it colonizes the R. equi genome, including the virulence plasmid. Despite its high mobility and transfer potential, the *erm*(46) element is confined to a specific R. equi clone, likely as a result of strong coselection due to concomitant chromosomal rifampin resistance.

## RESULTS

### R. equi *erm*(46) is carried on a conjugative plasmid.

**FIG 1:**
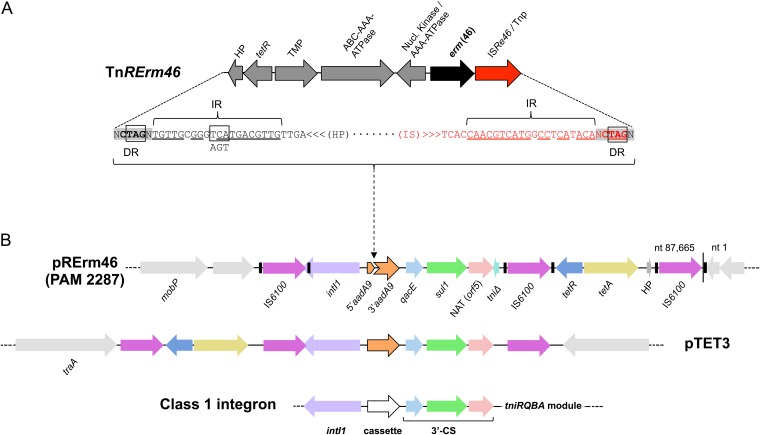
Integrative elements of the R. equi macrolide resistance plasmid pRErm46. (A) Genetic structure of the 6.9-kb transposon Tn*RErm46* carrying the macrolide resistance gene *erm*(46). The IS*Re46* transposase is a novel member of the IS*481* family. Its closest homolog is IS*Rae1* from Rhodococcus aetherivorans (amino acid identity, 82% [274/333]). TMP is a 25-kDa Gap-like (TauE/SafE superfamily) putative transmembrane protein with a possible role in small-molecule transport/export. See text and Table S1 for other Tn*RErm46* components. DR, direct repeats (shaded) at the junction with genomic DNA and adjacent inverted repeats (IR), which comprise the CTAG sequence targeted by Tn*RErm46*. This sequence provides the TAG stop codons for the IS*Re46* transposase on the right end and for some target genes—whose function is thus not interrupted (e.g., *parB* on the PAM 2287 pRErm46 plasmid)—on the left end. The left IR provides the TGA stop codon of the transposon’s distal hypothetical gene (HP). Stop codons are boxed. (B) Genetic structure of pRErm46’s class 1 integron (C1I). The black rectangles flanking IS*6100* represent the 14-bp terminal inverted repeats with the sequence GGCTCTGTTGCAAA. The Tn*RErm46* insertion within the C1I cassette gene *aadA9*, unique to PAM 2287’s pRErm46, is indicated. The *aadA9* gene is uninterrupted in most MLS^r^ isolates where pRErm46 was detected, typically within an ≈4.4-kb contig covering the *intI1*-*aadA9*-*qacE*-*sul1*-*orf5*-*tni*Δ C1I sequence. PAM 2287’s pRErm46 carries two additional Tn*RErm46* copies (see Fig. 3 and Table S2). nt, nucleotide.

Whole-genome sequences (WGS) of 18 MLS-resistant (MLS^r^) R. equi equine isolates previously used to identify the *erm*(46) gene (32) were analyzed to characterize the nature of the transferable element involved. In most WGS assemblies, *erm*(46) was found together with an adjacent integrase/transposase gene (32) in a unique 6.9-kb contig (Fig. 1A). In one instance (strain PAM 2275 [see Table S2 in the supplemental material]), the same *erm*(46)-containing 6.9-kb fragment was present in an ≈50- to 62-kb contig (depending on the assembly) with no homology to any known R. equi genomic DNA (21, 34) and which contained genes encoding putative conjugation-related type IV secretion system (T4SS) proteins plus an insertion sequence (IS*6100* [see below]) at one of its ends. We hypothesized this larger contig might be part of a conjugative plasmid harboring the *erm*(46) gene within a 6.9-kb transposable element, which then had moved to different genome locations, explaining its assembly as a separate 6.9-kb contig in most isolates.

10.1128/mBio.02260-19.4 TABLE S2 MLS^r^ and control MLS^s^ equine isolates from the United States analyzed in this study with indication of identified Tn*RErm46* insertion sites. Download Table S2, PDF file, 0.1 MB. Copyright © 2019 Álvarez-Narváez et al. 2019 Álvarez-Narváez et al. This content is distributed under the terms of the Creative Commons Attribution 4.0 International license.

10.1128/mBio.02260-19.3 TABLE S1 Annotation of pRErm46 from PAM 2287 (see Fig. 3 for the genetic structure of the plasmid). Download Table S1, PDF file, 0.2 MB. Copyright © 2019 Álvarez-Narváez et al. 2019 Álvarez-Narváez et al. This content is distributed under the terms of the Creative Commons Attribution 4.0 International license.

We next sought to ascertain the physical presence of *erm*(46)-associated extrachromosomal DNA in R. equi MLS^r^ plasmid preparations. We used isolates PAM 2287, which transferred *erm*(46) at frequencies of up to (1.59 ± 0.52) × 10^−3^, and PAM 2285, for which *erm*(46) transfer was not detected [transfer frequencies for the rest of the isolates were (7.41 ± 3.86) × 10^−5^ to (1.13 ± 0.31) × 10^−6^]. We also used two MLS^r^ transconjugants between PAM 2287 and an MLS-susceptible (MLS^s^) recipient devoid of any extrachromosomal DNA (103S^–^Hyg^r^, a virulence plasmid [pVAPA]-cured genome strain 103S with a chromosomal hygromycin resistance marker), namely, PAM 2350, which acquired *erm*(46) but not pVAPA, and PAM 2351, which acquired both *erm*(46) and pVAPA (see Materials and Methods).

**FIG 2:**
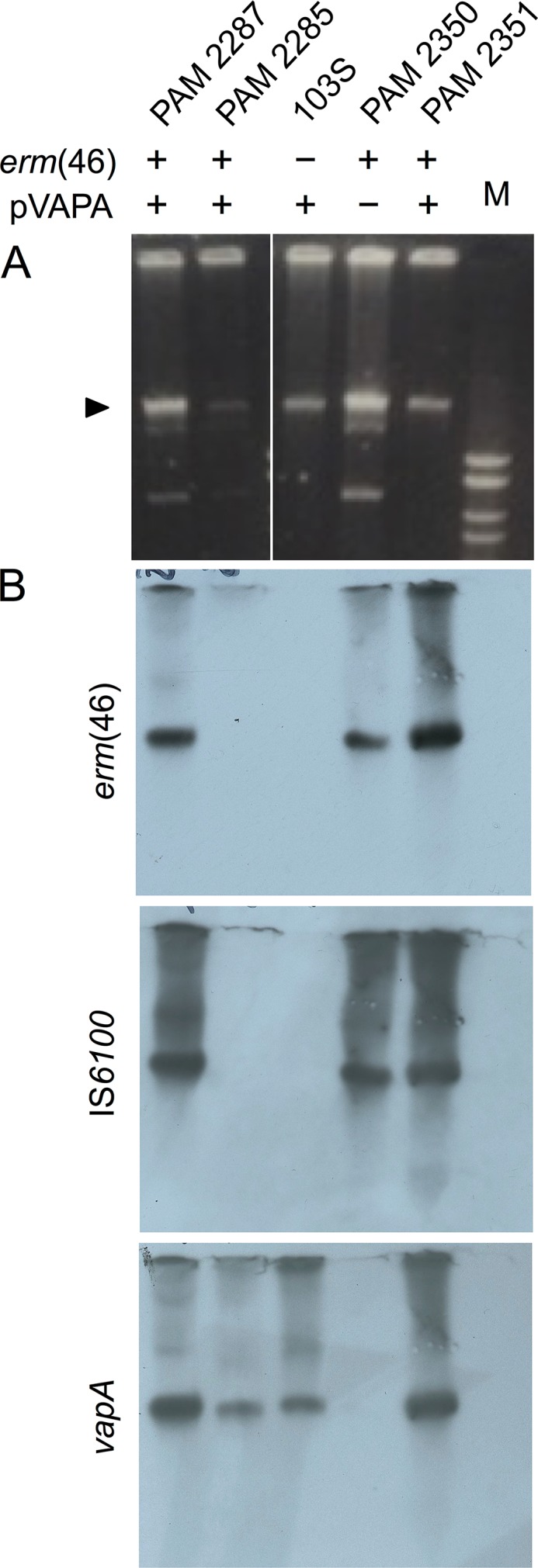
Identification of *erm*(46)-associated plasmid DNA (pRErm46). Presence and absence of the macrolide resistance gene *erm*(46) or the virulence plasmid is indicated by plus and minus signs, respectively. The macrolide-susceptible, virulence plasmid-harboring R. equi 103S strain was used as a control. (A) Agarose gel of plasmid preparations of the indicated representative strains. M, molecular size marker (Promega 1-kb DNA ladder; top band is 10 kb). The arrowhead indicates the position of the virulence plasmid and the (pRErm46) resistance plasmid band; both migrate at a theoretical height of ≈15 kb due to supercoiling. The additional faster-migrating band in the plasmid preparations of PAM 2287 and cognate PAM 2350 transconjugant corresponds to a cryptic plasmid that appears to be inconsistently transferred. (B) Corresponding Southern blots probed with *erm*(46), IS*6100*, and *vapA* (pVAPA virulence plasmid) probes (see text for details).

All *erm*(46)-positive strains showed the presence of a distinct plasmid band, strikingly, at the very same position as the plasmid DNA from MLS^s^ 103S even if lacking pVAPA (PAM 2350 transconjugant) (Fig. 2A). The identity of the DNA bands was determined by Southern blotting with specific probes for pVAPA (internal *vapA* fragment) and the larger ≈50-/62-kb contig [internal *erm*(46) and IS*6100* fragments]. The *vapA* probe identified the expected pVAPA band in the equine isolates PAM 2285 and 2287, transconjugant PAM 2351, and control 103S but did not hybridize with the similarly positioned plasmid DNA band from the *erm*(46)-positive PAM 2350 transconjugant (Fig. 2B). On the other hand, the *erm*(46) and IS*6100* probes detected a specific band at the same position as the *vapA* signal in isolate PAM 2287 as well as the PAM 2350 and 2351 transconjugants, but not the control 103S plasmid preparation (Fig. 2B). These data were consistent with *erm*(46) being present in an extrachromosomal element of a size similar to that of pVAPA also harboring IS*6100*, perhaps in several copies. Indeed, BLASTN searches identified the insertion sequence in 14 additional MLS^r^ genome assemblies (but not MLS^s^ genomes) as a discrete 881-bp contig exactly corresponding to the coding sequence of the 254-residue IS*6100* tranposase plus cognate 14-bp terminal inverted repeats (IR).

Interestingly, no *erm*(46) or IS*6100* probe signal was detected in PAM 2285 plasmid preparations (Fig. 2B) despite the 6.9-kb *erm*(46) contig being present in the WGS assemblies. Since *erm*(46) transfer was not observed with PAM 2285, we surmised that in this isolate the 6.9-kb *erm*(46)-containing putative transposable element might have jumped onto the chromosome with subsequent loss of the conjugative plasmid carrying it.

### Characterization of pRErm46, a self-transmissible plasmid responsible for emerging macrolide resistance in R. equi.

**FIG 3:**
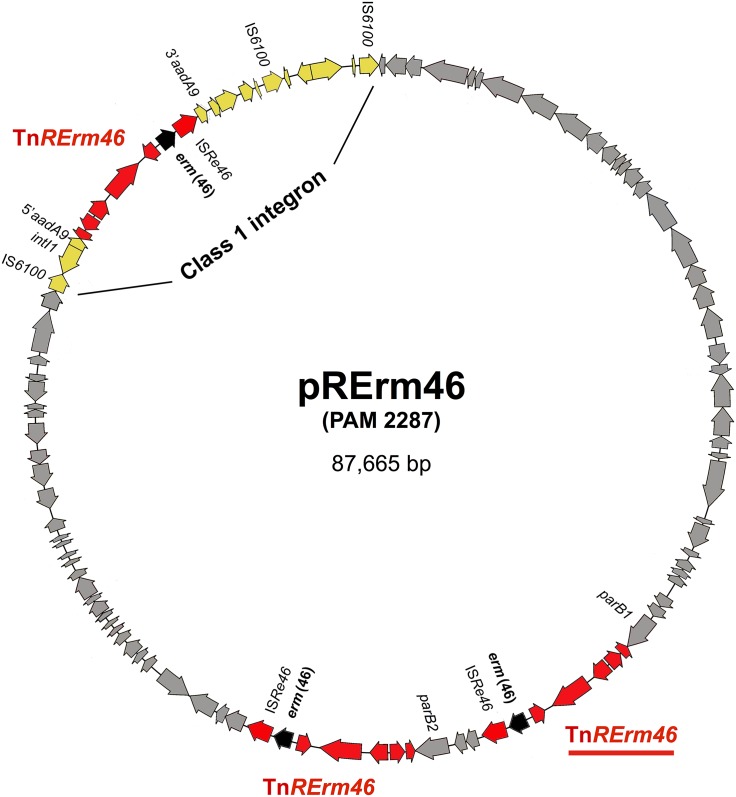
Genetic structure of pRErm46 from R. equi PAM 2287 (reference sequence, GenBank accession no. KY494640). Highlighted in color are the class 1 integron (yellow) and the Tn*RErm46* transposon (three copies, in red; underlined, Tn*RErm46* insertion shared by all pRErm46 plasmids, presumably the original site from which secondary transpositions took place). The macrolide resistance *erm*(46) gene within Tn*RErm46* is in black. Excluding the integrative elements, the pRErm46 backbone (in gray) is a conjugative replicon of 56.7 kb. pRErm46 (PAM 2287) sequence annotation is in Table S1.

Single-molecule real-time (SMRT) sequencing was performed on a plasmid-enriched DNA preparation from the pVAPA-negative transconjugant PAM 2350. This yielded the complete 5.0-Mb 103S chromosome and a second scaffold of 87,665 bp comprising the previously identified ≈50-/62-kb contig. This was assumed to correspond to the *erm*(46)-containing putative conjugative plasmid that comigrated with pVAPA (80.6 kb) and was named pRErm46 (Fig. 3).

pRErm46 is a covalently closed DNA molecule as determined with Circlator (35) and verified by PCR (Fig. 3). It shares no overall homology with database entries, except for discrete segments strongly similar to sequences from corynebacterial and (many) β-proteobacterial plasmids (see below). pRErm46 contains 104 coding DNA sequences (CDSs), of which 92% had homologs in other *Actinobacteria*, particularly *Rhodococcus* spp., suggesting a rhodococcal origin. The G+C content (65.09%) is similar to the average for R. equi genomic DNA (68.7%) (21, 34).

A function could be predicted for 49% of the CDSs. This included products involved in plasmid maintenance (Fic/Doc-like protein), self-replication (DnaK and DnaB homologs, putative DNA gyrase/topoisomerase) and partitioning (ParA and duplicated ParB homologs) (Table S1). Self-transmissibility is mediated by a MOB-P relaxase (36), MobC-like relaxase-accessory protein (RAP) (37), and T4SS apparatus with a TraG-like protein and VirB4-like ATPase (38). A LysM-like murein endopeptidase and a secreted cutinase (Table S1) may help the DNA translocation complex through the bacterial cell envelope. Actinobacterial cutinases have lipolytic/esterase activity (39) and in mycobacteriophages are thought to act as auxiliary LysB lysins aiding the penetration of the thick mycolic acid layer (40, 41). A cutinase has been shown to promote conjugation of the *R. fascians* virulence plasmid pFiD188 (42).

### Integrative elements of pRErm46.

The additional new sequence in the 87.6-kb SMRT scaffold revealed a class 1 integron of the “clinical” type (43), with an *intI1* integron-integrase gene, passenger gene (*aadA9* aminoglucoside adenyltransferase) (44), and 3′ conserved segment (3′ CS) comprising *qacE* (quaternary ammonium compound efflux pump), *sulI* (drug-resistant sulfonamide target, dihydropteroate synthase) and *orf5* sequences (Fig. 1B and Fig. 3). BLASTN searches identified class 1 integron homologs with virtually the same sequence and core genetic structure in the plasmids pTET3 from Corynebacterium glutamicum (44) and pLEW279a from *Corynebacterium* sp. strain L2-79-05 (45) as well as *Arthrobacter* sp. genomic DNA. Except for the *aadA9* cassette specific to a subset of homologous sequences, and the 6.9-kb *erm*(46) element inserted within the *aadA9* gene in pRErm46 from PAM 2350 (Fig. 1; see also below), entries with identical DNA segments (BLASTN E value, 0.0; >99.6% identity) were abundant among betaproteobacterial plasmids and genomic islands in public databases.

pRErm46’s class 1 integron carries three directly repeated copies of the IS*6* family insertion sequence IS*6100* (Fig. 1B and Fig. 3). First identified in Mycobacterium fortuitum (46), IS*6100* is widespread among a diversity of Gram-negative organisms, consistent with an active horizontal exchange of this element between different bacterial groups (47). In *Salmonella*, IS*6100* is found to be associated with the complex class 1 integron that constitutes the mobilizable multidrug resistance SG1 genomic island (48). Three IS*6100* copies are also found with a similar arrangement in the homologous regions of the pTET3 (Fig. 1B) and pLEW279a plasmids, suggesting a recent common origin for the *Corynebacteriales* mobile class 1 integrons.

Two of pRErm46’s IS*6100* copies flank the core integron sequence. At the left, IS*6100* is inserted into the *intI1* coding sequence (Fig. 1B), which was interpreted in pTET3 as causing the truncation of the *intI1* gene (44). However, analysis of the (identical) region in pRErm46 reveals that the *intI1* open reading frame (ORF) extends into the 3′ end of the convergent IS*6100* sequence in the opposite strand and may code for a full-length integrase. The right IS*6100* copy is next to a putative NTP-binding protein/transposon resolvase pseudogene at the end of *orf5* of the 3′ CS (Fig. 1B). This is likely the remnant of a Tn*402*-like transposon typically carried by “preclinical” class 1 integrons, aka the *tni*Δ module (49, 50), which IS*6100* presumably functionally replaces.

The third IS*6100* copy in pRErm46 is at the end of an additional module that extends to the right of the integron and harbors a putative *tetR*-*tetA* tetracycline efflux determinant. An identical IS*6100*-flanked module (BLASTN E value, 0.0; >99.9% identity) is found in pTET3 at the opposite (*intI1*) side of the integron core (Fig. 1B), in pLEW279a, and in *Trueperella (Corynebacterium) pyogenes* chromosomal DNA, suggesting that it is independently mobilizable. IS*6100* transposition has been experimentally demonstrated in *Actinobacteria*, including *Mycobacterium* (46, 51), *Streptomyces* (52), and *Corynebacterium* (44). Due to the replicative transposition mechanism of IS*6*-like elements, involving cointegrate resolution by homologous recombination (47), directly repeated flanking IS copies are formed favoring local rearrangements (48, 49). Together with transposition itself, such recombination-driven rearrangements may explain the different location of the *tetR*-*tetA* module in the otherwise identical class 1 integrons from pRErm46 and pTET3.

The pRErm46 plasmid sequence of the PAM 2350 transconjugant (originally from the MLS^r^ equine isolate PAM 2287, which we establish as the reference sequence for pRErm46; GenBank accession no. KY494640) carries in addition three copies of the 6.9-kb *erm*(46) element (Fig. 3).

### Tn*RErm46* transposon.

The Tn*RErm46* transposon is 6,919 bp in length and comprises seven ORFs encoding, in this order, a hypothetical protein, a TetR family transcriptional regulator, a 25-kDa Gap-like (TauE/SafE superfamily) membrane transporter, an ABC ATPase with a duplicated NTP-binding domain, a putative nucleotide kinase with an AAA ATPase motif, the *erm*(46) 23S rRNA methyltransferase, and an IS*481* family transposase (Fig. 1A and Table S1). Except for the hypothetical protein, homology searches suggest an actinobacterial origin, specifically rhodococcal. Maximum identity values of 82 to 89% with its closest homologs (from *Nocardia*/*Rhodococcus* spp.) indicate that the transposase is a novel IS*481* member, which we named IS*Re46*.

**FIG 4:**
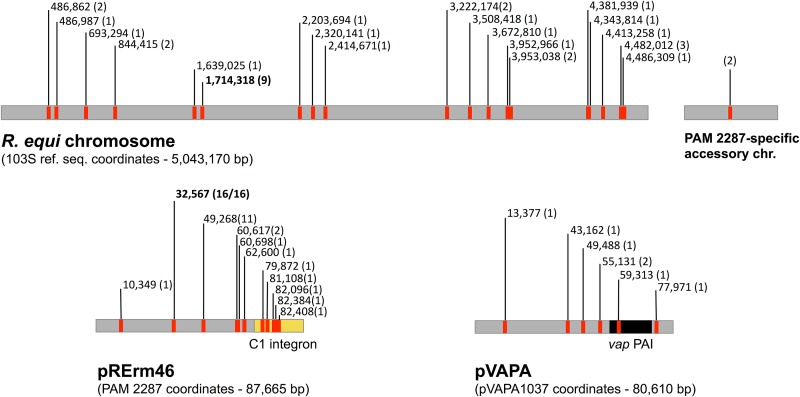
Tn*RErm46* insertions identified in pRErm46, the R. equi chromosome, and pVAPA virulence plasmid from the 18 MLS^r^ equine isolates analyzed in this study (cumulative). Numbers correspond to the insertion sites as per the sequence coordinates of the reference R. equi 103S chromosome (GenBank accession no. FN563149) (21), 103S pVAPA (pVAPA1037, GenBank accession no. AM947677) (8), and PAM 2287 pRErm46 (GenBank accession KY494640 [this study]). In parentheses is the number of instances in which a particular insertion was detected. In pRErm46, 16/16 corresponds to 100% of isolates in which pRErm46 sequences were detected in the genome assemblies. One of the 20 chromosomal insertion sites was in a resistant clone-specific region. The accessory genome accounts for ≈20% of an R. equi isolate’s gene content (34).

IS*481* family members are widely distributed in bacteria and may be associated, as in this case, with cargo genes such as antibiotic resistance determinants and regulators, constituting self-mobilizable transposons (53). IS*481* transposases belong to the Asp-Asp-Glu (DDE) catalytic motif type and are thought to operate through a copy-paste mechanism (53). The *erm*(46) element was confirmed as an IS*Re46*-driven transposable unit by the presence in all its multiple insertion sites (see below) of the directly repeated (DR) hexanucleotide NCTAGN, invariably followed inwards by the inverted repeat (IR) sequence 5′-TGTTGCGGGTCATGACGTTG-3′ (Fig. 1A). Analysis of the insertion sites in the R. equi genome (Fig. 4) indicates that CTAG is the target DNA sequence for transposition, of which the TAG triplet in the right flanking DR provides the UAG stop codon for the transposase (Fig. 1A). We named this novel macrolide resistance transposable element Tn*RErm46*.

One of the three Tn*RErm46* copies in pRErm46 from PAM 2350/2287 is inserted into the integron’s passenger gene (*aadA9*) (Fig. 1). The two other copies are found in a 16.7-kb region between positions 32562 at the 3′ end of the *parB* gene and 49273 before *parA* in pRErm46’s backbone (Fig. 3 and Table S1). In the left copy the IS*Re46* element is a pseudogene due to two frameshift mutations. The second transposition appears to have involved a duplication of the three left flanking genes from the plasmid backbone (pRErm46 CDS_0440, pRErm46 CDS_04450, and *parB*), which as a result are directly repeated between the two transposon copies (Fig. 3 and Table S1). In both Tn*RErm46* copies the TAG of the left NCTAGN DR provides the stop codon for the repeated *parB1* and *parB2* genes (Fig. 3). Interestingly, these transposition events took place at the *parA*/*B* plasmid partitioning module, previously identified in the R. equi virulence plasmids and related rhodococcal replicons as a hot spot for integration of foreign DNA (8, 13).

### Pervasive genomic colonization by Tn*RErm46*.

The multiple copies of Tn*RErm46* in pRErm46 (PAM 2350/2287) suggested that it is an actively transposable element. To gain insight into this issue, we searched for the Tn*RErm46* sequences in the WGS assemblies of the 18 MLS^r^ isolates. Contigs with 100% matching nucleotide sequences to either of the Tn*RErm46* ends were aligned with BLASTN against the R. equi chromosome and the reference sequences of the equine-type virulence plasmid pVAPA1037 (GenBank accession number AM947677) (8) and resistance plasmid pRErm46. To pinpoint the chromosomal insertions, we used a draft SMRT sequence of PAM 2287 (reference isolate of the MLS^r^ clone; see below) and the reference (complete) R. equi 103S genome sequence (21) for coordinate assignment.

Figure 4 shows the Tn*RErm46* insertions identified for all the MLS^r^ clinical isolates. Transposition into the bacterial chromosome was detected in all but one of the isolates (PAM 2275), up to five times in some cases. A total of 35 insertions at 20 different chromosomal sites were identified. In addition, Tn*RErm46* was found in the pVAPA virulence plasmid in four isolates (one copy in PAM 2283, 2284, and 2287 and four copies in PAM 2286). Except nucleotide position 55131 shared by pVAPA2286 and pVAPA2287, the element was inserted at different pVAPA locations (Fig. 4 and Table S2). Again, it is noteworthy that four of the seven pVAPA insertions were in the plamid’s replication/partitioning module. This region was previously identified in pVAPA and related circular rhodococcal plasmids as the integration site for the horizontal gene transfer (HGT) DNA which constitutes the plasmid-specific, niche-adaptive variable region (VR) (8, 13). In pVAPA, the VR corresponds to the horizontally acquired *vap* pathogenicity island (PAI), which itself provided one of the insertion sites for Tn*RErm46*. Consistent with the clinical source of the bacteria and the requirement of a functional virulence plasmid/*vap* PAI for host colonization (54, 55), none of the transposon insertions, including the one within the theoretical boundaries of the *vap* PAI (Fig. 4) (at nt position 59315 targeting the stop codon of a pseudogene), are likely to affect the expression of critical pVAPA genes, specifically virulence-associated determinants.

**FIG 5:**
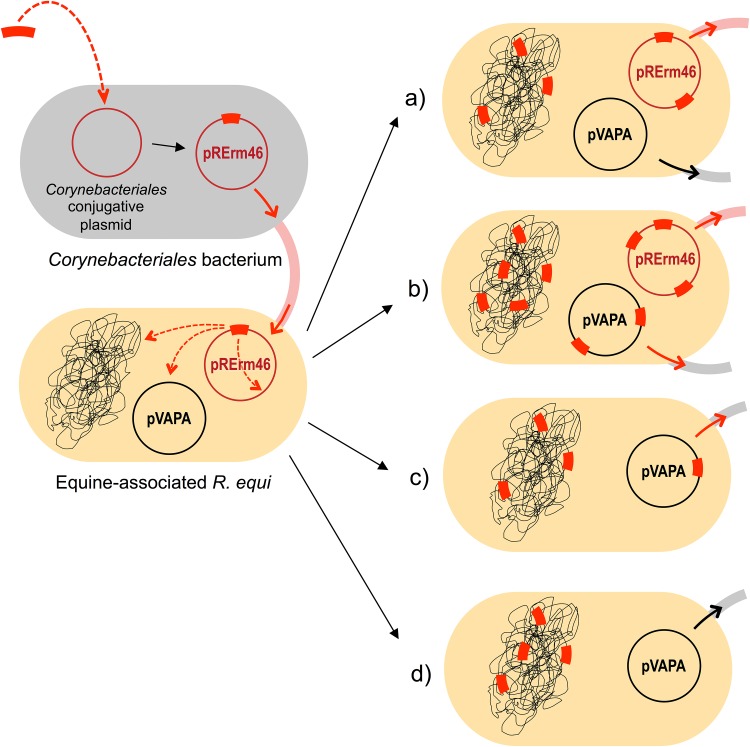
Fate of Tn*RErm46* macrolide resistance transposon upon acquisition by an equine R. equi isolate. (Top left) Formation of pRErm46 via transposition of Tn*RErm46* into a *Corynebacteriales* conjugative replicon; (bottom left) pRErm46 is conjugally transferred from a hypothetical *Corynebacteriales* donor to R. equi. The highly mobile Tn*RErm46* element can transpose from its original location (position 32567, common to all R. equi pRErm46 plasmids [see text and Fig. 4]) to other genome sites, resulting in several possible scenarios, as follows. (a) transposition within pRErm46 and onto the chromosome but not the virulence plasmid; (b) same as in panel a plus transposition to pVAPA; (c) same as in panel b, with subsequent loss of pRErm46; (d) same as in panel a, with subsequent loss of pRErm46. The pervasive colonization of the R. equi genome by Tn*RErm46* leads to stabilization of macrolide resistance in the host strain, while potential lateral transfer of Tn*RErm46* may occur via pRErm46, the pVAPA virulence plasmid, or both (solid red arrows). Options a, b, and d, in addition to Tn*RErm46* remaining in single copy at its original location in pRErm46 (isolate PAM 2275), have been verified in the 18 MLS^r^ equine isolates analyzed (see Table S2).

Finally, all pRErm46 plasmids harbored at least two Tn*RErm46* copies in a combination of common and unique locations. The only exception is PAM 2275, with a single transposon insertion at position 32567. This specific insertion is shared by all pRErm46 plasmids analyzed (Table S2), suggesting that it corresponds to the original transpositional event through which the pre-pRErm46 replicon acquired the *erm*(46) determinant (Fig. 5). Of note, the Tn*RErm46* insertion within the *aadA9* gene found in pRErm46 from PAM 2350/2287 (Fig. 1 and 3) is unique to this isolate. However, Tn*RErm46* was found within the integron’s 3′ CS in three other isolates, targeting *sul1* (once) and *orf5* (three different sites) (Table S2). Of a total of 11 identified insertion sites in the pRErm46 plasmids, five were within the class 1 integron (Fig. 4), consistent with bacterial integrons serving as flexible recruitment platforms for resistance-encoding DNA and transposable elements (43).

For three isolates, PAM 2280, 2285, and 2289, class 1 integron sequences were not found in the WGS assemblies despite the fact that the isolates carried the Tn*RErm46* element. However, pRErm46 (including the class 1 integron) was detected by PCR and BLASTN mapping of the Tn*RErm46* insertion sites in PAM 2289 and by PCR in PAM 2280 (Table S2), suggesting that perhaps the resistance plasmid was present in only a small fraction of these isolates’ populations. PAM 2285, in contrast, appears to have actually lost the pRErm46 plasmid and to carry Tn*RErm46* only on the chromosome (Table S2). This would explain the inability of this isolate to transfer the *erm*(46) element/MLS^r^ phenotype in mating experiments.

Overall, the data reveal a pattern of active, widespread dissemination of Tn*RErm46* across the host R. equi genome. This includes the extrachromosomal replicons, i.e., the pRErm46 resistance plasmid and the pVAPA virulence plasmid, both of which are conjugally transferable. Conjointly, all these mechanisms ensure the stable maintenance of the newly acquired resistance element in R. equi (Fig. 5).

### Cotransfer of macrolide resistance and virulence determinants.

In mating experiments with PAM 2287, 74% of the *erm*(46)-positive (erythromycin resistant [Erm^r^]) 103S^–^ transconjugants had also acquired the virulence plasmid. To further document that pVAPA can transfer *erm*(46), we examined one such *erm*(46)/pVAPA-positive transconjugant, PAM 2351 (see above), by SMRT sequencing. The sequence analyses confirmed that pVAPA from PAM 2351 carried Tn*RErm46* inserted at position 55132 (3′ end of *parB*) as in the donor PAM 2287, consistent with the transposon having been conjugally transferred *in situ* within pVAPA. Notably, this insertion was accompanied by three additional copies of Tn*RErm46* in the virulence plasmid (Fig. S1). These data confirm that the Tn*RErm46* element can actively transpose within a relatively short time frame. They also confirm that the virulence plasmid, essential for host colonization, may serve itself as a spread vector for the macrolide resistance determinant *erm*(46).

10.1128/mBio.02260-19.1 FIG S1 Tn*RErm46* insertions in pVAPA from PAM 2351 transconjugant (original donor, MLS^r^ equine isolate PAM 2287 [Table S2]). PAM 2351 has four Tn*RErm46* copies as determined by SMRT (PacBio) sequencing. One of them is immediately at the left of the original (position 55130) insertion and resulted from a secondary transposition into position 49488 downstream of pVAPA_0361 in the plasmid partitioning region. According to the plasmid scaffold assembly, this transposition was associated with the deletion of the left region of the original copy of Tn*RErm46* (encoding the hypothetical protein, TetR-like regulator, and membrane transporter) plus an adjacent 5,684-bp plasmid segment encompassing ORFs pVAPA_0370 to _0410. Another Tn*RErm46* copy was inserted at position 62049 at the 3′ end of ORF pVAPA_480 (*orf4*/*virR* virulence regulator) in the plasmid *vap* PAI (M. Letek, A. A. Ocampo-Sosa, M. Sanders, U. Fogarty, T. Buckley, D. P. Leadon, P. Gonzalez, M. Scortti, W. G. Meijer, J. Parkhill, S. Bentley, and J. A. Vázquez-Boland JA, J Bacteriol 190:5797–5805, 2008, https://doi.org/10.1128/JB.00468-08), the transposon’s DR providing the stop codon for the gene, thus not obviously affecting its functionality. Interestingly, this insertion was accompanied by a duplication of a portion of the *vap* PAI from pVAPA_0430 (*lsr2*) to pVAPA_480 (*orf4*/*virR*), where a third additional Tn*RErm46* insertion took place. Download FIG S1, PDF file, 0.3 MB. Copyright © 2019 Álvarez-Narváez et al. 2019 Álvarez-Narváez et al. This content is distributed under the terms of the Creative Commons Attribution 4.0 International license.

### Clonal spread of pRErm46/Tn*RErm46*.

R. equi isolates from equine settings or clinical specimens are genotypically diverse not only at a global population level (34) but also within the same farm (56) or even the same animal (56, 57). The various conjugal exchange routes for Tn*RErm46*, via either pRErm46, pVAPA, or both, hence would predict a situation of rapid spread across a diversity of R. equi strains. To investigate this, we performed a phylogenomic analysis of the 18 *erm*(46)-positive isolates available for this study and 27 control MLS^s^ strains. The resistant isolates were all obtained from different US states over a period of a decade (34) while the control susceptible set comprised six coetaneous random equine isolates from the same geographic areas (Table S2) plus 22 strains representative of the global genomic diversity of R. equi (34) (Table S3). The latter included the type strain DSM 20307 (=ATCC 6939) and 103S (= DSM 104936 = NCTC 13926) (21) used as the reference genome.

10.1128/mBio.02260-19.5 TABLE S3 Representative R. equi isolates used in this study. Genome assemblies were previously reported (E. Anastasi, I. MacArthur, M. Scortti, S. Alvarez, S. Giguere, and J. A. Vazquez-Boland, Genome Biol Evol 8:3140–3148, 2016, https://doi.org/10.1093/gbe/evw222). Download Table S3, PDF file, 0.1 MB. Copyright © 2019 Álvarez-Narváez et al. 2019 Álvarez-Narváez et al. This content is distributed under the terms of the Creative Commons Attribution 4.0 International license.

**FIG 6:**
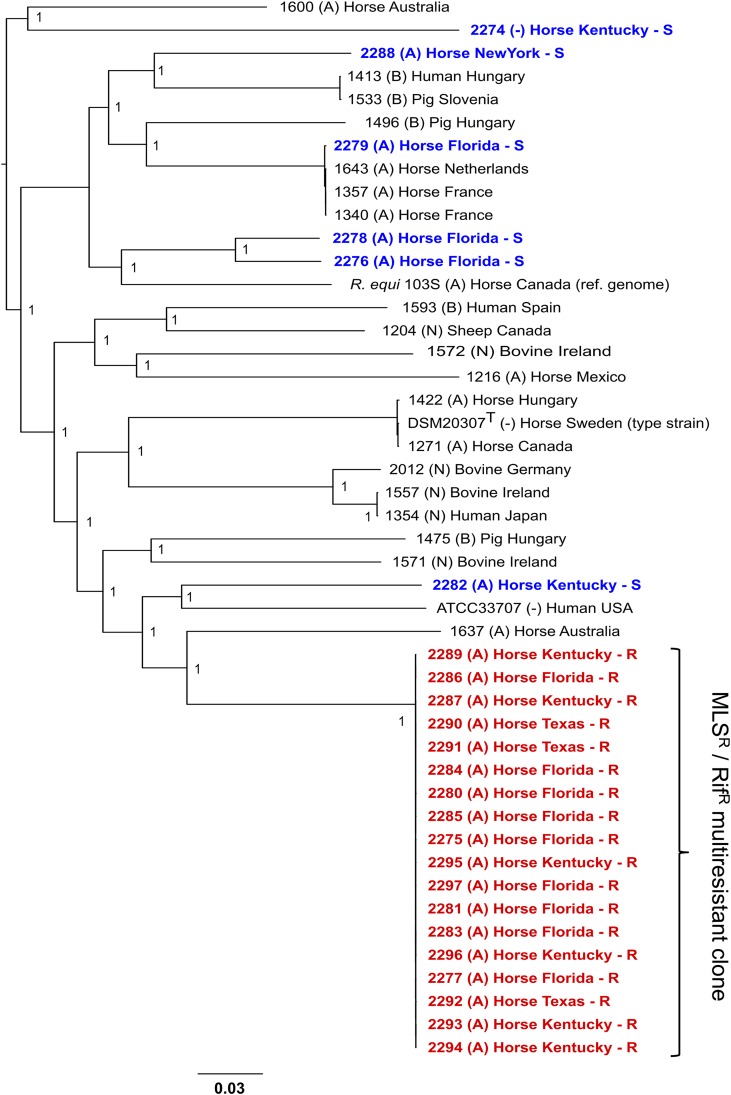
Clonal spread of Tn*RErm46*-mediated macrolide resistance. Phylogenetic tree of 46 R. equi isolates based on core genome SNP analysis using Parsnp (58). The genomes analyzed are from 18 MLS^r^ and 6 control MLS^s^ equine isolates from different U.S. states (highlighted in red and blue, respectively) plus 22 isolates representative of the genomic diversity of the species, including the reference genome from strain 103S (DSM 104936 = NCTC 13926; GenBank accession no. FN563149 [21]) and the type strain DSM 20307 (= ATCC 6939; GenBank accession no. LWTX00000000 [34]). Numbers in the nodes indicate bootstrap values for 1,000 replicates. The tree graph was constructed with FigTree (http://tree.bio.ed.ac.uk/software/figtree/). See also Fig. S2.

Figure 6 shows a core genome phylogeny based on a Parsnp alignment (58). While the control equine isolates were randomly distributed across the R. equi population structure, all 18 Tn*RErm46*-harboring isolates clustered together within a distinct clade at the top of the tree. In a context where all members of the R. equi species radiate at a short genetic distance from each other (34), the macrolide-resistant group differed from the nearest control isolates by ≈25,000 to 27,000 single nucleotide polymorphisms (SNPs) (over ≈3,800 core genes/4.2 Mbp). In contrast, the resistant group differed only by an average of 60 (43 to 102) SNPs in pairwise comparisons, indicating that it corresponds to a recently emerged R. equi clone (Fig. S2).

10.1128/mBio.02260-19.2 FIG S2 SNP analysis of the MDR R. equi 2287 clonal isolates (in red) and control susceptible equine isolates (in blue) against the prototype 2287 genome (highlighted in cyan). The reference R. equi genome strain 103S (M. Letek, P. González, I. Macarthur, H. Rodríguez, T. C. Freeman, A. Valero-Rello, M. Blanco, T. Buckley, I. Cherevach, R. Fahey, A. Hapeshi, J. Holdstock, D. Leadon, J. Navas, A. Ocampo, M. A. Quail, M. Sanders, M. M. Scortti, J. F. Prescott, U. Fogarty, W. G. Meijer, J. Parkhill, S. D. Bentley, and J. A. Vázquez-Boland, PLoS Genet 6:e1001145, 2010, https://doi.org/10.1371/journal.pgen.1001145) is boxed in cyan. See Fig. 6 and the text. SNPs are represented as purple vertical lines, and phylogenetic relationships are shown in a neighbor-joining tree. Note that the clonal isolates differ by only a few SNPs (43 to 102) compared to the >25,000-SNP difference between the representative macrolide-susceptible isolates. SNP visualization was achieved by the Gingr program of genomes aligned with Parsnp in the Harvest suite (T. J. Treangen, B. D. Ondov, S. Koren, and A. M. Phillippy, Genome Biol 15:524, 2014, https://doi.org/10.1186/s13059-014-0524-x). Download FIG S2, PDF file, 0.3 MB. Copyright © 2019 Álvarez-Narváez et al. 2019 Álvarez-Narváez et al. This content is distributed under the terms of the Creative Commons Attribution 4.0 International license.

The *erm*(46)-positive R. equi bacteria also exhibited rifampin resistance (Rif^r^) (MIC_90_ >32 μg/ml, versus 0.25 μg/ml for MLS^s^ equine isolates) (32, 59). Analysis of the *rpoB* sequences revealed in all of them the same missense mutation at codon 443 resulting in a Ser→Phe substitution. None of the susceptible isolates carried any *rpoB* mutation. The location corresponds to Escherichia coli RpoB position 531 within rifampin resistance-determining region 1 (RRDR-1; residues 507 to 533), where the same substitution is known to cause the Rif^r^ phenotype (60). Different RRDR-1 Rif^r^ mutations have been previously reported for R. equi, including Ser531Leu and Ser531Trp (E. coli numbering), associated with MICs of 8 μg/ml and >64 μg/ml, respectively (19, 28–30). Like Ser531Trp, the Ser531Phe *rpoB* mutation, which had never been described before for R. equi, introduces a large aromatic side chain at position 531 and is also associated with a relatively high level of rifampin resistance.

Our data thus indicate that the emerging macrolide resistance detected among R. equi equine isolates in the United States is due to a specific clone that acquired the Tn*RErm46* element and is characterized by a distinct Rif^r^ mutation.

### Fitness neutrality of pRErm46.

We finally considered to what extent the clonal spread of *erm*(46)-mediated macrolide resistance could have been influenced by a specific adaptation of the pRErm46 plasmid to the host strain. To explore this, we tested whether pRErm46 acquisition entailed any fitness cost upon transfer to the 103S strain, which belongs to an early clonal bifurcation of R. equi (lineage I) different from that of the resistant clone (lineage II) (34). The analyses were performed with low-passage-number PAM 2350 cultures stored after conjugation to minimize the potential impact of adaptive plasmid cost amelioration (61, 62).

**FIG 7:**
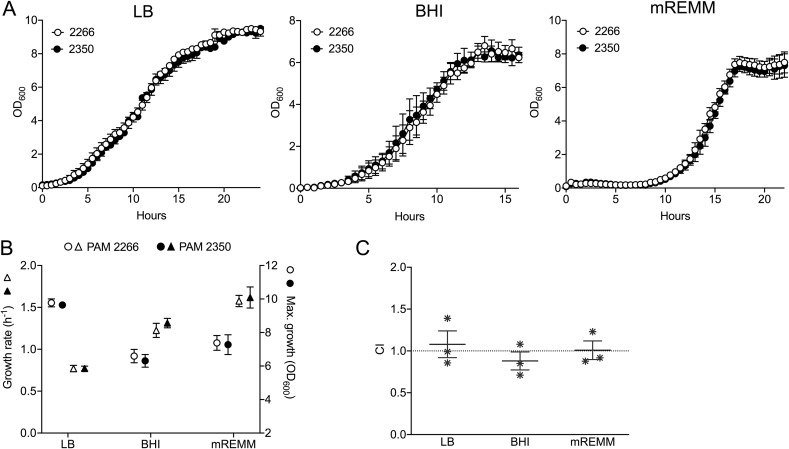
Acquisition of pRErm46 is fitness neutral. pRErm46 was conjugally transferred from the MDR clone (PAM 2287) to a different chromosomal lineage (strain 103S [see Fig. 6]). PAM 2350, pRErm46 103S transconjugant. PAM 2266, isogenic recipient 103S bacteria without pRErm46 (virulence plasmid-cured 103S with a chromosomal Hyg^r^ marker for counterselection of pRErm46 donor strain in mating experiments). (A) Growth curves in rich complex medium (LB or BHI) and chemically defined medium (mREMM [see Materials and Methods]). (B) Growth rate (μ) and maximum growth (A) values from experiments in panel A. (C) Competitive growth experiments. No significant differences between the isogenic pair of bacteria with and without pRErm46 were observed (all *P* values ≥ 0.7, one-way ANOVA followed by Šidák *post hoc* comparison).

No significant differences in exponential growth rate (μ) or maximum growth (A) were observed between pRErm46-bearing PAM 2350 and the plasmid-devoid 103S recipient in either complex media (brain heart infusion [BHI] and Luria-Bertani [LB] broth) or chemically defined medium (Fig. 7A and B). The two strains also showed no significant differences in competitive ability *in vitro* in the absence of antibiotic pressure (Fig. 7C). Thus, pRErm46 does not seem to impose significant fitness costs upon transfer to a different R. equi genomic background.

## DISCUSSION

The rise of antimicrobial resistance (AMR) among leading human pathogens is often associated with specific clonal complexes that become globally disseminated (63). While phylogenomic studies have clearly linked the emergence of such successful clades to founder genetic events involved in the acquisition of resistance (63–68), the drivers of the clonal expansion remain largely speculative. Our study captured one such founder event at the early stages of its evolution in a major animal pathogen, R. equi, providing valuable insight into the determinants of AMR clonality.

We show that the emerging *erm*(46)-mediated MLS^r^ detected among equine R. equi isolates in the United States (32) is due to a specific clone that carries a mobile element and a chromosomal mutation conferring dual resistance to, respectively, macrolides and rifampin, the mainstay of antibiotic therapy against foal rhodococcosis since the 1980s (20, 26). Diverse Rif^r^ *rpoB* mutations have been previously reported for R. equi (19, 28–30), indicating that such chromosomal mutations occur regularly and alone are insufficient to support the clonal expansion of a specific strain. It seems likely that the conjugal acquisition of the hitherto undescribed pRErm46 plasmid and cognate *erm*(46)-carrying Tn*RErm46* transposon has been the decisive trigger in the emergence of the R. equi multidrug-resistant (MDR) clone.

Mobile resistance elements have been traced to the origin of major MDR clonal complexes (63). The elements often become stabilized in the host genome and evolve with the clone, with little evidence of active, short-term mobilization or lateral transferability. Examples include SCC*mec* in methicillin-resistant Staphylococcus aureus (MRSA) (69), the AbaR genomic island/composite transposon in Acinetobacter baumannii clonal complex GC1 (68, 70), and the SG1 MDR island in Salmonella enterica serovar Typhimurium DT104 (71). On other occasions, exemplified by the plasmid-borne genes *bla*_KPC_ in Klebsiella pneumoniae clonal group 258 and *bla*_CTX-M-15_ in Escherichia coli ST131 (66, 67, 72), the mobile resistance element promotes the clonal amplification while at the same time it exploits the dominance of their host clone to disseminate and drive the expansion of other diverse bacterial lineages/species (63).

In our case, the *erm*(46) macrolide resistance determinant is restricted to a specific R. equi clone yet its vehicle is a highly mobile, potentially actively transferable element. Moreover, transferability is maximized through co-option of the conjugative machinery of the pVAPA virulence plasmid (Fig. 5), physically linking two essential traits required for the colonization of a macrolide-rifampin-treated equine host. Furthermore, evidence indicates that pVAPA plasmids are actively exchanged between R. equi lineages (9, 34), facilitated by alternation of spontaneous plasmid loss in the absence of host pressure (7, 11) and high-frequency conjugative reacquisition (12). While these features theoretically anticipate the rapid spread of Tn*RErm46* across the array R. equi populations typically colonizing the equine farm environment (56, 57), the MDR clone (dubbed 2287 after the isolate from which the pRErm46 reference sequence derives) has been circulating since at least 2002 (Table S2), until now with no apparent Tn*RErm46* spillover to other R. equi lineages.

How can the paradoxical clonal confinement of the otherwise highly mobile Tn*RErm46* element/pRErm46 plasmid be explained? Our data appear to exclude fitness constraints, because acquisition of the pRErm46 plasmid does not seem to entail significant costs in a different R. equi genomic background. In addition, any fitness cost would be largely compensated in antibiotic-treated animals in which resistance confers a selective advantage (73). We suggest that clonality is primarily determined by the inability of pRErm46/Tn*RErm46* to ensure alone the evolutionary viability of the host R. equi bacteria in the absence of a chromosomal *rpoB* mutation under combined macrolide-rifampin pressure. Under these conditions, the probability of concurrent acquisition of the pRErm46 plasmid and a Rif^r^ mutation reoccurring *de novo* would be vanishingly small (as low as ≈10^−10/–14^, considering individual *in vitro* frequencies of, respectively, ≈10^−2/–6^ [most often 10^−5^] and ≈10^−8^ [30; our unpublished observations]) compared to the likelihood of the existing R. equi 2287 clone spreading through horse trading or cross-exposure at racecourses.

Human-pathogenic MDR clones typically begin as localized expansions and rapidly—within a few years—disseminate pandemically and diversify in clonal complexes (63–66, 72). Despite the high prevalence of R. equi in horse breeding countries, after more than 15 years R. equi 2287 still appears to be at the initial stages of local dissemination. This apparently slow expansion arguably stems from the fewer opportunities for spread afforded by the significantly smaller size and restricted geographical mobility of the equine versus human populations. Bar the diversity of Tn*RErm46* insertion sites, R. equi 2287 isolates also show very limited genetic variability, presumably a reflection of the ecological stability of the equine niche to which the clone is restricted. We predict that, over time, R. equi 2287 will probably become globally disseminated, accumulate additional resistance mechanisms (63, 64, 68, 72, 74), and eventually enter a path of fast genome evolution fueled by the continued transposition and amplification of the IS*Re46* element, as observed in Bordetella pertussis with the homologous insertion sequence IS*481* (75). pRErm46 (or Tn*RErm46* carried by other mobile elements, e.g., bacteriophages [41]) is also likely to be horizontally transferred to other R. equi strains or equine-associated microbiota.

In addition to being a veterinary proxy of the nosocomial setting in terms of intensive antimicrobial use and close-contact transmission, the particularities of R. equi antibiotic control in equine farms, based on the same drug combination for decades, offer a unique “controlled” scenario that helps illuminating the factors underlying AMR clonal spread. Our findings illustrate that under sustained combination therapy, AMR requires the co-occurrence of independent founder events, thereby effectively reducing the likelihood of resistance emergence and expansion to a specific bacterial clone. They also highlight the impact of ecological bottlenecks imposed by antimicrobial use in shaping the population dynamics of pathogens (74) and the potential effectiveness of combination therapies in limiting not only the emergence (76, 77) but also, critically, the spread of resistance—even if conferred by highly mobile elements.

While consubstantial to and underpinning AMR, clonal spread provides at the same time a predictable framework to develop control strategies. The pRErm46 plasmid and Tn*RErm46* element here reported, together with the identification of the Rif^r^ 2287 lineage harboring these, lay the foundations for the effective tracking and control of emerging macrolide-rifampin multiresistance among equine R. equi isolates and its potential zoonotic transmission to humans.

## MATERIALS AND METHODS

### Bacteria, culture conditions, and reagents.

The 18 MLS^r^ and 6 control MLS^s^ isolates analyzed in this study were selected from a collection of R. equi cultures obtained from tracheobronchial aspirates or postmortem tissue of infected foals from different U.S. states between 2002 and 2013 (32) (Table S2). WGS shotgun assemblies thereof were previously used in the identification of the *erm*(46) gene (32). WGS assemblies from 22 additional control isolates representative of the global diversity of R. equi have been previously described (34). The presence of the virulence plasmid and pRErm46 plasmid/Tn*RErm46* element was routinely checked by PCR using specific oligonucleotide primers (Table S4). R. equi was grown in brain heart infusion medium (BHI; Difco-BD) at 30°C unless otherwise stated, with orbital shaking (200 rpm) for fluid cultures. Bacteriological agar (Oxoid) was used at 1.6% (wt/vol) for plate cultures. Antibiotic supplements were added as required after autoclaving. Chemicals and oligonucleotide primers were purchased from Sigma-Aldrich unless otherwise stated.

10.1128/mBio.02260-19.6 TABLE S4 Main oligonucleotides used in this study. Download Table S4, PDF file, 0.1 MB. Copyright © 2019 Álvarez-Narváez et al. 2019 Álvarez-Narváez et al. This content is distributed under the terms of the Creative Commons Attribution 4.0 International license.

### Conjugal transfer of pRErm46.

Mating experiments were carried out as described by Anastasi et al. (32) using a 1:1 donor to recipient ratio. A virulence plasmid (pVAPA)-cured derivative of the genome strain 103S (21) with a chromosomal hygromycin resistance marker was used as a recipient for counterselection of the wild-type donor isolate (designated 103S^–^ Hyg^r^; internal collection no. PAM 2266). After incubation at 30°C for 72 h, transconjugants were selected on BHI agar supplemented with 200 μg/ml of hygromycin B and 10 μg/ml of erythromycin. pRErm46 transfer was confirmed by PCR analysis of at least 30 Hyg^r^ Erm^r^ colonies per experiment using *ad hoc* primers (Table S4). Transfer frequencies were calculated by dividing the number of Hyg^r^ Erm^r^ bacteria by the total number of recipient bacteria.

### DNA techniques.

Plasmid DNA for Southern blotting was extracted from exponential BHI cultures (optical density at 600 nm [OD_600_] ≈1.0) using an alkaline precipitation protocol as previously described (7). Total and plasmid-enriched DNA for sequencing was obtained using the GenElute bacterial genomic DNA kit (Sigma-Aldrich) and QIAfilter midikit (Qiagen), respectively. Total DNA for routine PCR tests was prepared by heating single bacterial colonies at 100°C in 100 μl of ultrapure water and centrifugation for 90 s at 16,000 × *g*. PCR was performed as previously described (13). Oligonucleotide primers used are shown in Table S4. For Southern blotting, plasmid preparations were electrophoresed in 0.5% agarose and transferred to positively charged nylon membranes (Roche) after treatment of the gels with denaturing solution (1.5 M NaCl 0.5 N NaOH) for 30 min and neutralizing solution (1.5 M NaCl, 0.5 M Tris-HCl pH 7.5) twice for 20 min. Probes consisted of digoxigenin-labeled internal fragments of the genes of interest [*vapA*, *erm*(46), and IS*6100* transposase] generated with the PCR DIG probe synthesis kit (Roche) using suitable oligonucleotide primers (Table S4). Membranes were hybridized at high stringency and developed according to the manufacturer’s instructions. Hybridized membranes were stripped with 0.2 N NaOH–0.1% SDS at 45°C for 30 min before reprobing.

### DNA sequencing and analysis.

Illumina shotgun libraries of 24 R. equi equine clinical isolates (18 MLS^r^ and six MLS^s^) obtained by Anastasi et al. (32) were used in this study. Genomes were assembled *de novo* as previously described (34) or using a modified protocol with Cutadapt software for adapter removal (78) and QUAST for quality assessment (79). SMRT PacBio genome sequencing of PAM 2350 and 2351 transconjugants was performed by BGI Genomics (Hong Kong) and BaseClear sequencing services (The Netherlands), respectively. Minimap and Miniasm (*de novo* assembler) software was used for mapping and *de novo* assembling single SMRT data (80), and Racon for consensus calling (81). Circlator (35) was run with error corrected reads from the Canu v1.3 error correction module for assembly circularization to obtain an accurate linear representation of circular sequences (82). Annotation of the assemblies was performed using Prokka V1.11 (83) and InterProScan v5.17. pRErm46 annotations were manually inspected and curated based on Blast analysis. Whole-genome phylogenies were inferred using Parsnp in the Harvest suite, designed for SNP analysis between closely related species/strains (≥97% average nucleotide identity [ANI]) (58). Parsnp uses FastTree 2 (84) to build approximate maximum likelihood trees from core-genome SNPs.

### Fitness assays.

Effects on bacterial fitness were investigated by monitoring the growth and competitive ability in two complex media (BHI and LB) and in chemically defined medium (modified R. equi minimal medium [mREMM]) (34) supplemented with two rapidly metabolizable carbon sources (50 mM sodium lactate and 25 mM sodium acetate), as previously described (85). Briefly, exponential-phase BHI cultures were washed in phosphate-buffered saline (PBS), resuspended in fresh medium to give an OD_600_ of ≈0.05, and triplicate 400-μl aliquots of the suspension transferred to 48-well plates. Bacterial growth was monitored at 30°C every 30 min using an automated plate reader (Optima apparatus, BMG). The maximum growth rate (μ) and maximum bacterial cell density (A) were estimated from spline-fits of OD_600_ values using the Grofit package in R (86) as previously described (85). Pairwise competition experiments were carried out as described above with a bacterial inoculum consisting of a 1:1 mix of the two test bacteria. At specific time points, cultures were sampled and appropriate dilutions spread on LB agar supplemented with suitable antibiotics for differential counting. The competitive index was calculated using the formula CI = (test/reference log CFU ratio at *t* = n)/(test/reference log CFU ratio at *t* = 0) (13, 85).

### Statistics.

Growth parameters were analyzed using one-way analysis of variance (ANOVA) followed by Šidák *post hoc* multiple-comparison tests unless otherwise stated. One‐sample Student *t*‐tests were used to determine if competition index (CI) values differed significantly from 1 (the theoretical CI value if the ratio of the competing strains remains the same with respect to that at time zero). Statistical analyses were performed using Prism 8.0.2 software (GraphPad Software Inc., San Diego, CA).

### Accession number(s).

The reference sequence of the pRErm46 plasmid from R. equi PAM 2287 has been deposited at GenBank under accession no. KY494640. R. equi WGS assemblies used in this study that were not previously reported have been deposited under accession no. MULU00000000, MUMB00000000, MUMA00000000, MULW00000000, MULT00000000, MULY00000000, MULZ00000000, MULX00000000, MUXK00000000, MVDS00000000, MVDT00000000, MVDU00000000, MVDV00000000, MVDQ00000000, MVDR00000000, MUXJ00000000.
