# Supplementary material for: Clonal Confinement of a Highly Mobile Resistance Element Driven by Combination Therapy in Rhodococcus equi
Source: mBio. 2019 Oct 15;10(5):e02260-19. doi: 10.1128/mBio.02260-19 (PMC6794481; doi:10.1128/mBio.02260-19)
Supplement: TABLE S2 [file mBio.02260-19-st002.pdf]

Table S2. MLS^R and control MLS^S equine isolates from the US analyzed in this study with indication of identified Tn*RErm46* insertion sites.

PAM no. [a]	Isolation year	Origin	Macrolide & Rifampin	WGS assembly accession no.	pRErm46 PCR [b]	Tn*RErm46* insertions [c]		
						pRErm46	Chromosome	pVAPA
2274	2011	Kentucky	S	LWTQ00000000	–	—	—	—
2275	2003	Florida	R	MULU00000000	+	32,567	—	—
2276	2001	Florida	S	LWTR00000000	–	—	—	—
2277	2004	Florida	R	MUMB00000000	+	32,567 / 82,408 (C1I [d] *orf5*)	1,714,318	—
2278	2002	Florida	S	MUMA00000000	–	—	—	—
2279	2001	Florida	S	LWTS00000000	–	—	—	—
2280	2009	Florida	R	MULW00000000	+	—	486,862 / 844,415 / 3,222,174 / 4,482,012 / 1 in clone's accessory chr. seq.	—
2281	2005	Florida	R	MULT00000000	+	32,567 / 49,268	1,714,318	—
2282	2011	Kentucky	S	LWTT00000000	–	—	—	—
2283	2002	Florida	R	MULY00000000	+	32,567 / 49,268	1,714,318 / 4,413,318	13,377
2284	2005	Florida	R	MULZ00000000	+	32,567 / 49,268 / 60,698 / 82,096 (C1I *orf5*)	3,508,418 / 3,672,810 / 4,381,939 / 4,482,012	49,488
2285	2005	Florida	R	LWTU00000000	Only Tn*RErm46*	—	486,862 / 844,415 / 3,222,174 / 4,482,012 / 1 in clone's accessory genome	—
2286	2005	Florida	R	MULX00000000	+	32,567 / 62,600	4,486,309	43,162 / 55,131 / 59,313 / 77,971
2287	2010	Kentucky	R	LWTV00000000	+	32,567 / 49,268 / 79,872 (C1I *aadA9*)	—	55,131
2288	2010	New York	S	LWTW00000000	–	—	—	—
2289	2010	Kentucky	R	MUXK00000000	+	32,567 / 49,268	693,294 / 4,343,814	—
2290	2010	Texas	R	MUXK00000000	+	32,567 / 49,268 / 60,617	3,952,966	—

Table S2 (cont.).

PAM no. [a]	Isolation year	Origin	Macrolide & Rifampin	WGS assembly accession no.	pRErm46 PCR [b]	Tn*RErm46* insertions		
						pRErm46	Chromosome [c]	pVAPA
2291	2010	Texas	R	MVDS00000000	+	32,567 / 49,268 / 60,617	1,639,025 / 2,414,671	—
2292	2010	Texas	R	MVDT00000000	+	10,349 / 32,567 / 82,384 (C1I *orf5*)	1,714,318	—
2293	2011	Kentucky	R	MVDU00000000	+	32,567 / 49,268	1,714,318 / 3,953,038	—
2294	2011	Kentucky	R	MVDV00000000	+	32,567 / 49,268	1,714,318 / 3,953,038	—
2295	2011	Kentucky	R	MVDQ00000000	+	32,567 / 81,018 (C1I *sul1*)	1,714,318 / 2,203,694 / 2,320,141	—
2296	2001	Kentucky	R	MVDR00000000	+	32,567 / 49,268	486,978 / 1,714,318	—
2297	2002	Florida	R	MUXJ00000000	+	32,567 / 49,268	1,714,318	—

[a] JV-B laboratory isolate collection.

[b] Presence of pRErm46 plasmid and Tn*RErm46* element was analysed by PCR using six sets of oligonucleotide primers targeting: *erm*(46) gene, IS*Re46* transposase, IS*6100* from pRErm46's class 1 integron, and four genes of pRErm46 backbone (*traG*, *mobC*, *lysM* and *parA*; see Table S1). A "+" sign indicates positivity to all six PCR reactions. See Table S4.

[c] Coordinates according to reference sequences for pRErm46 plasmid (from PAM 2287, this study) and 103S chromosome and pVAPA virulence plasmid (refs. 8, 21). See Fig. 4.

[d] C1I, class 1 integron.
