# Supplementary material for: Clonal Confinement of a Highly Mobile Resistance Element Driven by Combination Therapy in Rhodococcus equi
Source: mBio. 2019 Oct 15;10(5):e02260-19. doi: 10.1128/mBio.02260-19 (PMC6794481; doi:10.1128/mBio.02260-19)
Supplement: TABLE S1 [file mBio.02260-19-st001.pdf]

Table S1. Annotation of pRErm46 from PAM 2287 (see Fig. 3 for the genetic structure of the plasmid).

pRERm46 – locus tag	Location (nt positions)	Size (bp)	Product	Gene
0010	1-238	237	Hypothetical protein	HP
0020	280-1086	807	Conserved hypothetical protein in *Rhodococcus* sp.	HP
0030	1098-1796	699	Conserved hypothetical protein in *Rhodococcus* sp.	HP
0040	1793-3595	1803	TraG conjugal transfer protein, TraG/TraD /VirD4 family homolog, present in other rhodococci and *Actinobacteria*	*traG*
0050	3645-3914	270	Conserved hypothetical protein in *Rhodococcus* sp.	HP
0060	3911-4198	288	Conserved hypothetical protein in *Rhodococcus* sp.	HP
0070	4195-5940	1746	Conjugation protein Type IV secretory pathway, VirB4 component	*virB4*
0080	5970-7460	1491	Conserved hypothetical protein in *Rhodococcus* sp. and *Nocardi*a sp.	HP
0090	7525-9033	1509	Conserved hypothetical protein in *Rhodococcus* sp. and *Nocardi*a sp.	HP
0100	9027-9893	867	Conserved hypothetical protein in *Rhodococcus* sp. and *Actinobacteria*	HP
0110	9919-10569	651	Conserved hypothetical protein in *Rhodococcus* sp. and *Actinobacteria*	HP
0120	10622-10888	267	Conserved hypothetical protein in *Rhodococcus* sp. and *Actinobacteria*	HP
0130	10896-11189	294	Conserved hypothetical protein in *Rhodococcus* sp. and *Actinobacteria*	HP
0140	11201-11875	675	Conserved hypothetical protein in *Rhodococcus* sp. and *Actinobacteria*	HP
0150	11872-12465	594	Conserved hypothetical protein in *Rhodococcus* sp. and *Actinobacteria*	HP
0160	12462-14138	1677	LysM/M23 murein DD-endopeptidase. Present in multiple species of *Rhodococcu*s and *Nocardia*	*lysM*
0170	14225-15880	1656	Conserved hypothetical protein in *Rhodococcus* sp. and *Nocardia* sp. with cutinase domain	cutinase
0180	15901-16755	855	Conserved hypothetical protein in *Rhodococcus* sp. and *Nocardi*a sp.	HP
0190	16765-17706	942	Conserved hypothetical protein in *Rhodococcus* sp. and *Nocardi*a sp.	HP
0200	17780-19000	1221	Conserved hypothetical protein in *Rhodococcus* sp. DNA translocase FtsK domain. Putative conjugation protein	HP
0210	19307-20080	774	XRE-family transcriptional regulator	TR
0220	20077-20493	417	Hypothetical protein	HP
0230	20480-21832	1353	NADPH-P450 reductase	P450
0240	21859-22992	1134	Conserved hypothetical protein in *Rhodococcus* sp. and *Nocardi*a sp.	HP
0250	22989-23495	507	Conserved hypothetical protein in *Rhodococcus* sp. and *Actinobacteria*	HP
0260	23724-23945	222	Conserved hypothetical protein in *Rhodococcus* sp. and *Actinobacteria*	HP
0270	24246-25943	1698	Conserved hypothetical protein in *Rhodococcus* sp. and *Actinobacteria*. Metal binding domain, Lrp HTH domain	HP
0280	26460-26660	201	Pseudogene	
0290	26726-27703	978	Conserved hypothetical protein in *Rhodococcus* sp. and *Actinobacteria* with DUF	HP
0300	27851-28102	252	Pseudogene	
0310	28282-28674	393	Conserved hypothetical protein in *Rhodococcus* sp. and *Actinobacteria*	HP
0320	28763-29044	282	Conserved hypothetical protein in *Rhodococcus* sp.	HP

Table S1 (cont.).

pRERm46_ locus tag	Location (nt positions)	Size (bp)	Product	Gene
0330	29044-29355	312	Putative membrane protein	MP
0340	30001-30453	453	Conserved hypothetical protein in *Rhodococcus* sp. and *Actinobacteria*	HP1 R1
0350	30507-30941	435	Conserved hypothetical protein in *Rhodococcus* sp.	HP2 R1
0360	31098-32567	1470	ParB-like nuclease domain protein	*parB1*
0370	32578-32943	366	Hypothetical protein	HP
0380	32992-33594	603	Bacterial regulatory protein	*tetR*
0390	33693-34415	723	Sap, sulfolipid-1-addressing protein	TMP
0400	34767-36461	1695	Putative ABC transporter AAA-ATPase	AAA-ATPase
0410	36824-37384	561	Nucleotide kinase / AAA-ATPase	Nucl. kinase/ AAA-ATPase
0420	37670-38482	813	Macrolide resistance 23S rRNA methyltransferase	*erm*(46)
0430	38479-39093	615	IS*481* family transposase	IS*Re46*
0440	39793-40245	453	Conserved hypothetical protein in *Rhodococcus* sp. and *Actinobacteria*	HP1 R2
0450	40297-40731	435	Conserved hypothetical protein in *Rhodococcus* sp.	HP2 R2
0460	41015-42358	1344	ParB-like nuclease domain protein	*parB2*
0470	42369-42734	366	Hypothetical protein	HP
0480	42783-43385	603	TetR family transcriptional regulator	tetR
0490	43484-44206	723	Sap, sulfolipid-1-addressing protein	TMP
0500	44558-46252	1695	Putative ABC transporter AAA-ATPase	AAA-ATPase
0510	46615-47175	561	Nucleotide kinase / AAA-ATPase	Nucl. kinase/ AAA-ATPase
0520	47461-48273	813	Macrolide resistance 23S rRNA methyltransferase	*erm*(46)
0530	48270-49271	1002	IS*481* family transposase	IS*Re46*
0540	49493-50302	810	ParA partitioning protein	*parA*
0550	50280-50705	426	Conserved hypothetical protein in *Rhodococcus* sp.	HP
0560	50799-51956	1158	Conserved membrane protein in *Rhodococcus* sp.	MP
0570	52021-53406	1386	Transmembrane pentapeptide repeat protein conserved in *Actinobacteria*	TMP
0580	53928-54272	345	Conserved hypothetical protein in *Actinobacteria*	HP
0590	54438-54704	267	Hypothetical protein	HP
0600	54765-55313	549	Conserved hypothetical protein in *Actinobacteria*	HP
0610	55442-55783	342	Conserved hypothetical protein in *Actinobacteria*	HP
0620	55913-56104	192	Putative DNA-binding protein (HTH containing)	HP
0630	56278-56445	168	Conserved hypothetical protein in *Rhodococcus* sp.	HP
0640	56642-56866	225	Thioredoxin/Glutaredoxin-like protein	*trx*
0650	56898-57437	540	Replicative DNA helicase DnaB	*dnaB*

Table S1 (cont.).

pRERm46_ locus tag	Location (nt positions)	Size (bp)	Product	Gene
0660	57503-57724	222	Conserved hypothetical protein in *Actinobacteria*	HP
0670	57721-58593	873	Fic/Doc-like protein (plasmid maintenance)	*fic/doc*
0680	58620-58928	309	Conserved hypothetical protein in *Rhodococcus* sp.	HP
0690	59229-59393	165	Conserved hypothetical protein in *Rhodococcus* sp. and *Nocardia* sp.	HP
0700	59559-59717	159	Hypothetical protein	HP
0710	60063-60218	156	Hypothetical protein	HP
0720	60298-60387	90	Hypothetical protein	HP
0730	60727-61224	498	Conserved hypothetical protein in *Rhodococcus* sp.	HP
0740	61654-62355	702	Conserved hypothetical protein in *Actinobacteria*	HP
0750	62589-63485	897	Conserved hypothetical protein in *Rhodococcus* sp. and *Actinobacteria*	HP
0760	63499-64065	567	Serine recombinase family	*res/inv*
0770	64327-65160	834	Gyrase / topoisomerase	*gyr/top*
0780	65412-65723	312	Conserved hypothetical protein in *Rhodococcus* sp.	HP
0790	65750-65968	219	Hypothetical protein	HP
0800	66061-66849	789	Conserved hypothetical protein, Abi-like CAAX protease domain (self-immumnity)	HP
0810	66853-67194	342	DnaK/Hsp70 chaperone	*dnaK*
0820	67819-68217	399	MobC relaxase accessory protein (RAP)	*mobC*
0830	68214-69974	1761	MOB-P relaxase	*mobP*
0840	70025-70771	747	Conserved hypothetical protein in *Rhodococcus* sp.	HP
0850	70811-71575	765	IS*6*-family insertion sequence IS*6100*	IS*6100*
0860	71392-72555	1164	Integron integrase / XerC/D superfamily site-specific recombinase	*intI1*
0861	72554-72968	414	Aminoglycoside 3”-adenyltransferase, 5’ fragment	5’*aadA9*
0870	72970-73335	366	Hypothetical protein	HP
0880	73384-73986	603	TetR family transcriptional regulator	*tetR*
0890	74085-74807	723	Sap, sulfolipid-1-addressing protein	IMP
0900	75159-76853	1695	Putative ABC transporter AAA-ATPase	AAA-ATPase
0910	77216-77776	561	Nucleotide kinase / AAA-ATPase	Nucl. kinase/ AAA-ATPase
0920	78062-78874	813	Macrolide resistance 23S rRNA methyltransferase	*erm*(46)
0930	78871-79872	1002	IS*481* family transposase	IS*Re46*
0940	80058-80450	393	Aminoglycoside 3”-adenyltransferase, 3’ fragment	3'*aadA9*
0950	80614-80961	348	Quaternary ammonium compound efflux pump / multidrug transporter EmrE	*qacE*
0960	80955-81758	804	Drug-resistant sulfonamide target, dihydropteroate synthase	*sul1*
0970	81922-82422	501	Putative N-acetyltransferase family enzyme	*orf5 /* NAT

Table S1 (cont.).

pRERm46_ locus tag	Location (nt positions)	Size (bp)	Product	Gene
0980	82405-82542	138	Putative NTP-binding protein / transposon resolvase pseudogene	*tni*Δ
0990	82929-83693	765	IS*6*-family insertion sequence IS*6100*	IS*6100*
1000	83784-83969	186	Hypothetical protein	HP
1010	84332-84901	570	Tetracycline repressor protein class A	*tetR*
1020	84904-86163	1260	Tetracycline resistance protein, class C	*tetA*
1030	86596-86697	102	Hypothetical protein	HP
1040	86883-87647	765	IS*6*-family insertion sequence IS*6100*	IS*6100*
