# Supplementary material for: Clonal Confinement of a Highly Mobile Resistance Element Driven by Combination Therapy in Rhodococcus equi
Source: mBio. 2019 Oct 15;10(5):e02260-19. doi: 10.1128/mBio.02260-19 (PMC6794481; doi:10.1128/mBio.02260-19)
Supplement: FIG S1 [file mBio.02260-19-sf001.pdf]

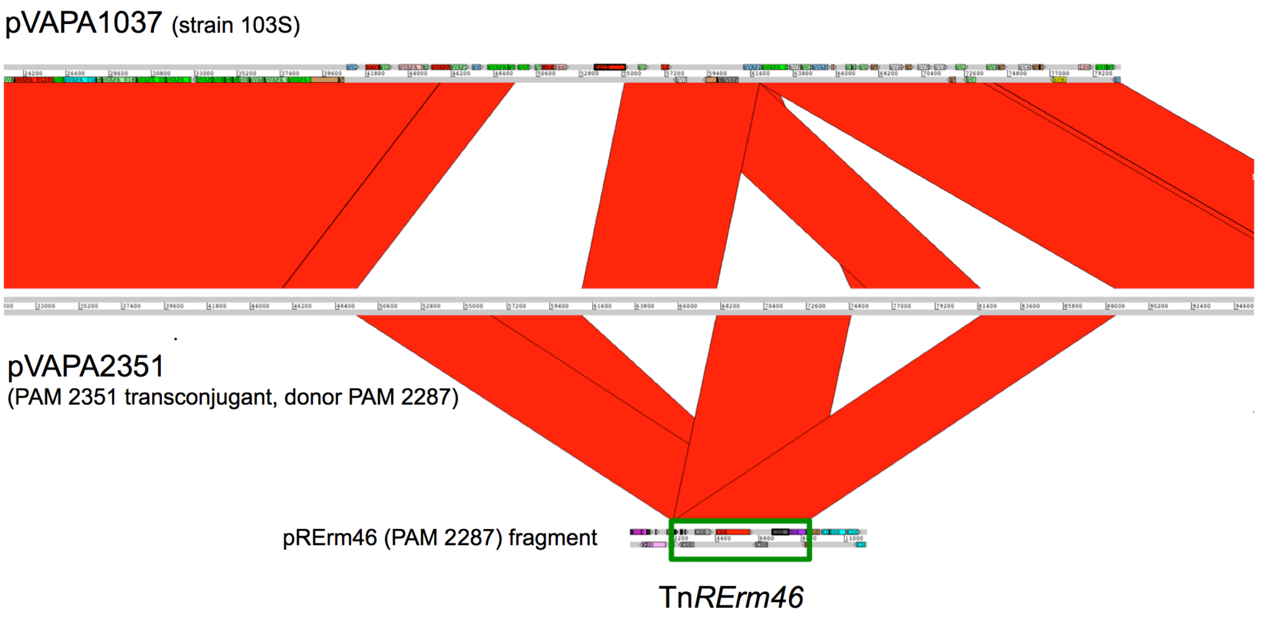

FIG S1. Tn*RErm46* insertions in pVAPA from PAM 2351 transconjugant (original donor, MLS^R equine isolate PAM 2287, Table S2). PAM 2351 has four Tn*RErm46* copies as determined by SMRT (PacBio) sequencing. One of them is immediately at the left of the original (position 55,130) insertion and resulted from a secondary transposition into position 49,488 downstream of pVAPA_0361 in the plasmid partitioning region. According to the plasmid scaffold assembly, this transposition was associated with the deletion of the left region of the original copy of Tn*RErm46* (encoding the hypothetical protein, TetR-like regulator and membrane transporter) plus adjacent 5,684-bp plasmid segment encompassing ORFs pVAPA_0370 to _0410. Another Tn*RErm46* copy was inserted at position 62,049 at the 3' end of ORF pVAPA_480 (*orf4*/*virR* virulence regulator) in the plasmid *vap* PAI (8), the transposon's DR providing the stop codon for the gene, thus not obviously affecting its functionality. Interestingly, this insertion was accompanied by a duplication of a portion of the *vap* PAI from pVAPA_0430 (*lsr2*) to pVAPA_480 (*orf4*/*virR*), where a third additional Tn*RErm46* insertion took place.
