# Supplementary material for: Clonal Confinement of a Highly Mobile Resistance Element Driven by Combination Therapy in Rhodococcus equi
Source: mBio. 2019 Oct 15;10(5):e02260-19. doi: 10.1128/mBio.02260-19 (PMC6794481; doi:10.1128/mBio.02260-19)
Supplement: TABLE S3 [file mBio.02260-19-st003.pdf]

Table S3. Representative *R. equi* isolates used in this study. Genome assemblies previously reported in ref. (34).

PAM no. [a]	Original designation / public repository number	Source	Origin	pVAP type [b]	Accession nos.	Reference
1126	103S [c] (NCTC 13926, DSM 104936)	Horse	Canada	A	FN563149 RefSeq NC_014659.1, GCA_000196695.1	Letek et al., 2010 (21)
2298	DSM 20307[T d] (ATCC 6939[T], NCTC 1621[T])	Horse	Sweden	(-)	LWTX00000000	Anastasi et al., 2016 (34)
—	ATCC 33707 [e]	Human	Canada	(-)	RefSeq NZ_CM001149.1, GCA_000164155.2	-
1271	ATCC 33701 [f]	Horse	Canada	A	LWIC00000000	Anastasi et al., 2016 (34)
1204		Sheep	Canada	N	LWBN00000000	Anastasi et al., 2016 (34)
1216		Horse	Mexico	A	LWHS00000000	Anastasi et al., 2016 (34)
1340		Horse	France	A	LWHT00000000	Anastasi et al., 2016 (34)
1354		Human	Japan	N	LWHU00000000	Anastasi et al., 2016 (34)
1357		Horse	France	A	LWHV00000000	Anastasi et al., 2016 (34)
1413		Human	Hungary	B	LWHW00000000	Anastasi et al., 2016 (34)
1422		Horse	Hungary	A	LWHX00000000	Anastasi et al., 2016 (34)
1475		Pig	Hungary	B	LWHY00000000	Anastasi et al., 2016 (34)
1496		Pig	Hungary	(-) [g]	LWHZ00000000	Anastasi et al., 2016 (34)
1533		Pig	Slovenia	B	LWIA00000000	Anastasi et al., 2016 (34)
1557		Bovine	Ireland	N	LWIB00000000	Anastasi et al., 2016 (34)
1572		Bovine	Ireland	N	LXFI00000000	Anastasi et al., 2016 (34)
1600		Horse	Australia	A	LXFG00000000	Anastasi et al., 2016 (34)
1637		Horse	Australia	A	LWHR00000000	Anastasi et al., 2016 (34)
1643		Horse	Netherlands	A	LWTP00000000	Anastasi et al., 2016 (34)
2012		Bovine	Germany	N	LWTY00000000	Anastasi et al., 2016 (34)
1571		Bovine	Ireland	N	LWTO00000000	Anastasi et al., 2016 (34)
1593		Human	Spain	B	LXFH00000000	Anastasi et al., 2016 (34)

Footnotes to Table S3.

[a] JV-B laboratory isolate collection.

[b] Virulence plasmid type: A, equine-associated pVAPA; B, swine-associated pVAPB; N, ruminant-associated pVAPN; (-) no virulence plasmid. See ref. (9).

[c] Low passage clone of J.F. Prescott's strain 103 used to determine the complete (reference) genome sequence of *R. equi*. See ref. (21).

[d] Type strain of *R. equi* from German DSMZ culture collection. Other designations: ATCC 6939^T, ATCC 25729^T, CIP 53.72^T, NCTC 1621^T, NBRC 14956^T, NBRC 101255^T, NRRL B-16538^T, CCT 0541^T.

[e] Also known as J.F Prescott's isolate 80, draft genome determined by the Human Microbiome project.

[f] Widely used together with strain103/103S as model strain in *R.equi* studies.

[g] Originally positive to pVAPB when tested at the time of inclusion in our laboratory PAM isolate collection.
