# Supplementary material for: Clonal Confinement of a Highly Mobile Resistance Element Driven by Combination Therapy in Rhodococcus equi
Source: mBio. 2019 Oct 15;10(5):e02260-19. doi: 10.1128/mBio.02260-19 (PMC6794481; doi:10.1128/mBio.02260-19)
Supplement: FIG S2 [file mBio.02260-19-sf002.pdf]

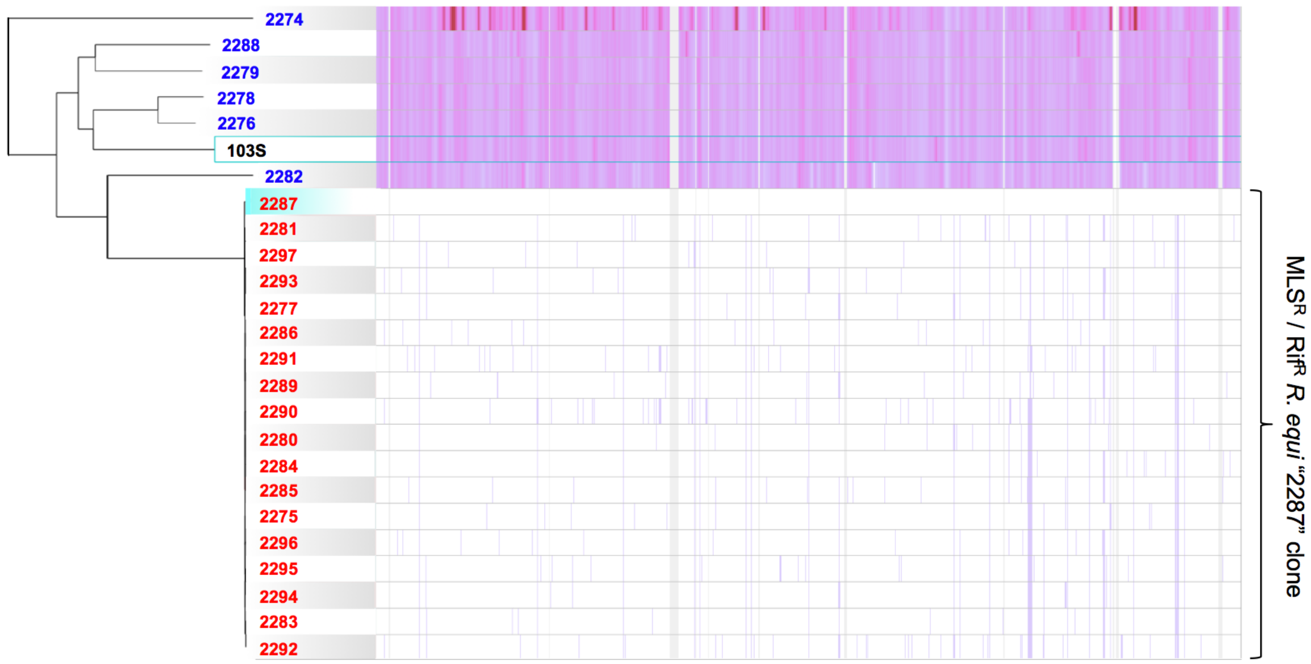


**FIG S2**. SNP analysis of MDR *R. equi* "2287" clonal isolates (in red) and control susceptible equine isolates (in blue) against the prototype 2287 genome (highlighted in cyan). Reference *R. equi* genome strain 103S (21) is boxed in cyan. See see Fig. 6 and text. SNPs are represented as purple vertical lines and phylogenetic relationships are shown in a neighbor-joining tree. Note that the clonal isolates differ by only a few SNPs (43 to 102) compared to the >25,000 SNPs difference between the representative macrolide-susceptible isolates. SNP visualization by Gingr program of genomes aligned with Parsnp in the Harvest suite (58).

.
