# Supplementary material for: Clonal Confinement of a Highly Mobile Resistance Element Driven by Combination Therapy in Rhodococcus equi
Source: mBio. 2019 Oct 15;10(5):e02260-19. doi: 10.1128/mBio.02260-19 (PMC6794481; doi:10.1128/mBio.02260-19)
Supplement: TABLE S4 [file mBio.02260-19-st004.pdf]

Table S4. Main oligonucleotides used in this study.

Primer	Sequence 5´ → 3´	Use	Reference
ChoE-1F<br>ChoE-1R	GCGCAACTACTTCGAGGAG<br>TTGTCGATTCCCATCGTC	*choE* chromosomal marker for confirmation of *R. equi*	Valero-Rello *et al*. 2015 (13)
TraA-F1<br>TraA-R1	AGAGTTCATGCGTGACAACG<br>GTCCACAGGTCACCGTTCTT	*traA* virulence plasmid backbone marker. Detection of *R. equi* virulence plasmid.	Ocampo-Sosa *et al.* 2007 (7)
IP1<br>IP2	GACTCTTCACAAGACGGT<br>TAGGCGTTGTGCCAGCTA	pVAPA virulence plasmid detection (*vapA* gene).	Ocampo-Sosa *et al.* 2007 (7)
erm(46)F<br>erm(46)R	TATGGAGTCGATCTGCAACG<br>GAGATCGGACGAGTCTGACA	*erm*(46) macrolide resistance gene detection.	This study
lysM-F<br>lysM-R	GGGCATACGAGAAGAGAGTT<br>CAAGGTCTACTCCACCGTGT	pRErm46 backbone detection (*lysM* gene)	This study
traG-F<br>traG-R	ACCGTCGTAGCAGTAGCC<br>CCTCAGCGAGTGTCTTCTC	pRErm46 backbone detection (*traG* gene)	This study
parA-F<br>parA-R	GGTCTCCTGACTTCTGGTCT<br>CATGGCCTCATACAACTAGG	pRErm46 backbone detection (*parA* gene)	This study
mobC-F<br>mobC-R	GACCTGACTCCAACTTAGGG<br>AAGAGCGCTCCATAGAAGTC	pRErm46 backbone detection (*mobC* gene)	This study
28F<br>28R	GCTGCTGCGAAATGGTGGTT<br>AGGGTGATGTGATCCTGTGG	Detection of pRErm46 class I integron (IS*6100*)	This study
10F<br>10R	AATGGGTGCACCGCTACC<br>TGTATGAGGCCATGACGTTG	Tn*RErm46* transposon detection (IS*Re46*)	This study
